# Case report: Primary immunodeficiency due to a novel mutation in CARMIL2 and its response to combined immunomodulatory therapy

**DOI:** 10.3389/fped.2022.1042302

**Published:** 2023-01-16

**Authors:** Yu Zhu, Lili Ye, Hua Huang, Xuemei Xu, Yu Liu, Jian Wang, Yanliang Jin

**Affiliations:** ^1^ Department of Rheumatology & Immunology, Shanghai Children’s Medical Center, Shanghai Jiao Tong University, School of Medicine, Shanghai, China; ^2^ Department of Medical Genetics and Molecular Diagnostic Laboratory, Shanghai Children's Medical Center, Shanghai Jiao Tong University, School of Medicine, Shanghai, China

**Keywords:** primary immunodeficiency, novel mutation, combined immunotherapy, whole exome sequencing (WES), CD4+/CD8+ lymphocytes

## Abstract

Capping protein regulator and myosin 1 linker 2 (CARMIL2) is necessary for invadopodia formation, cell polarity, lamellipodial assembly, membrane ruffling, acropinocytosis, and collective cell migration. CARMIL2 deficiency is a rare autosomal recessive disease characterized by dysfunction in naïve T-cell activation, proliferation, differentiation, and effector function and insufficient responses in T-cell memory. In this paper, we report a 9-year-old female patient with a novel pathogenic variant in CARMIL2 (c.2063C > G:p.Thr688Arg) who presented with various symptoms of primary immunodeficiencies including recurrent upper and lower respiratory infections, perioral and perineum papules, reddish impetiginized atopic dermatitis, oral ulcer, painful urination and vaginitis, otitis media, and failure to thrive. A missense mutation leading to insufficient CARMIL2 protein expression, reduced absolute T-cell and natural killer cell (NK cell) counts, and marked skewing to the naïve T-cell form was identified and indicated defective maturation of T cells and B cells. Following 1 year of multitargeted treatment with corticosteroids, hydroxychloroquine, mycophenolate mofetil, and thymosin, the patient presented with significant regression in rashes. CD4+ T-cell, CD8+ T-cell, and NK cell counts were significantly improved.

## Introduction

Capping protein regulator and myosin 1 linker 2 (CARMIL2) belongs to the human CARMIL family and encodes a 1,435 amino acid protein. It has been reported that CARMIL2 is necessary for invadopodia formation, cell polarity, lamellipodial assembly, membrane ruffling, acropinocytosis, and cell migration (1–3). CARMIL2 acts as a molecular link between vimentin filaments and dynamic actin assembly (1, 4, 5) and promotes actin polymerization at the leading edge of migrating cells (1, 6). In addition, CARMIL2 is involved in CD28-mediated T-cell costimulation and activation (5, 7–11).

CARMIL2 deficiency is a rare autosomal recessive disease characterized by dysfunction in naïve T-cell activation, proliferation, differentiation, and effector function and insufficient responses in T-cell memory (5, 8, 11, 12). The clinical manifestations of CARMIL2 deficiency are recurrent infections (mainly respiratory). Skin features include skin warts, verrucous papules, eczematous dermatitis, psoriatic rash, seborrheic dermatitis, recurrent condyloma, solar urticaria, and spongiotic dermatitis. Patients with CARMIL2 deficiency are sometimes prone to Epstein–Barr virus (EBV)-related smooth muscle tumors. Other clinical features include lymphocytic esophagitis, dysphagia, Crohn's disease, and failure to thrive. The clinical presentations of CARMIL2 deficiency that have been previously reported are summarized in Supplementary Table S1 (5, 10, 12–20).

In this paper, we report a 9-year-old female patient, born to Chinese consanguineous parents, who presented with recurrent respiratory infections, persistent dermatitis, recurrent skin abscess, oral ulcer, otitis media, and failure to thrive. Whole exome sequencing was performed in samples from the girl and her parents and revealed an unreported missense variant in the CARMIL2 gene (NM_001013838.2:c.2063C>G:p.Thr688Arg) that was predicted to be deleterious to the patient. A heterozygous mutation in CARMIL2 was also identified in each parent, who were asymptomatic carriers. By analyzing the clinical manifestations and immunological characteristics of this patient, recognition of CARMIL2 deficiency is expanded, underscoring the importance of consideration of molecular causes in patients with primary immunodeficiencies (PID).

## Materials and methods

### Clinical case

The patient and her family members were recruited from Shanghai Children's Medical Center. Gastroenterological endoscopy was performed in July 2020 to evaluate the patient's gastrointestinal inflammation in the Digestive Department, and a lung biopsy was performed in August 2020 in the General Surgery Department due to the patient's recurrent respiratory infections. The patient was diagnosed with PID based on genetic analysis and treated in the Rheumatology Department, and therapeutic efforts were observed for2 years, starting in 2020.

### Genetic workup

Genomic DNA was extracted from peripheral blood isolated from the patient and her family members to perform exome sequencing, following standard instructions. The whole exome sequencing was performed at the Clinical Molecular Diagnostic Laboratory at Shanghai Children's Medical Center.

### Western blotting

Proteins in blood samples taken from the patient and her family members were extracted and analyzed by Western blotting using standard protocols. An anti-CARMIL2 antibody (NBP2-62215; EM-53 NOVUS, United States) was used to detect CARMIL2 protein levels, and antibeta actin (mAbcam 8226; Abcam, United States) was used as a loading control. Results were analyzed using FlowJo V10.

### 3D modeling

A three-dimensional homology structure of the leucine-rich repeat (LRR) domain was modeled using SWISS-MODEL software.

### Flow cytometry

Peripheral blood mononuclear cells (PBMCs) were incubated with directly labeled antibodies to detect cell surface proteins. 4′6-Diamidino-2-phenylindole (DAPI) was used to exclude nonviable cells. Positive staining was considered based on the negativity of isotype control. Antibodies used included antihuman CD4 FITC (BioLegend, United States), antihuman CD8 Percp-cy5.5 (BioLegend, United States), antihuman CD45 RA-PE (BioLegend, United States), antihuman CCR7 BV421 (BioLegend, United States), antihuman CD19 Percp-cy5.5 (BioLegend, United States), antihuman CD27 PE-cy7 (BioLegend, United States), and antihuman IgD FITC (BioLegend, United States).

### Ethical considerations

Clinical information and biospecimens from the patient and her family members were obtained upon written consent.

## Results

### Clinical features of the patient

The patient was a 9-year-old girl, born to Chinese consanguineous parents, who presented with recurrent and intermittent upper and lower respiratory infections since early childhood. Her childhood respiratory infectious problems greatly improved with age. The patient complained of recurrent perioral and perineum asymmetrical erythematous papules and oral ulcers since 2018, which were alleviated by usual treatment. In addition, she had recurrent symptoms of frequent, urgent, and painful urination along with vaginal discomfort, which was diagnosed as recurrent urethritis and vaginitis. In addition, she has erythematous impetiginized atopic dermatitis that was widespread on her hands, fingers, and feet, perionyxis on her fingers and toes, and plantar heel pain. The cutaneous and mucosal infections were associated with multiple symptoms, including a hoarse voice, otitis media, and short stature with a height of less than 95% since childhood.

**Figure 1:**
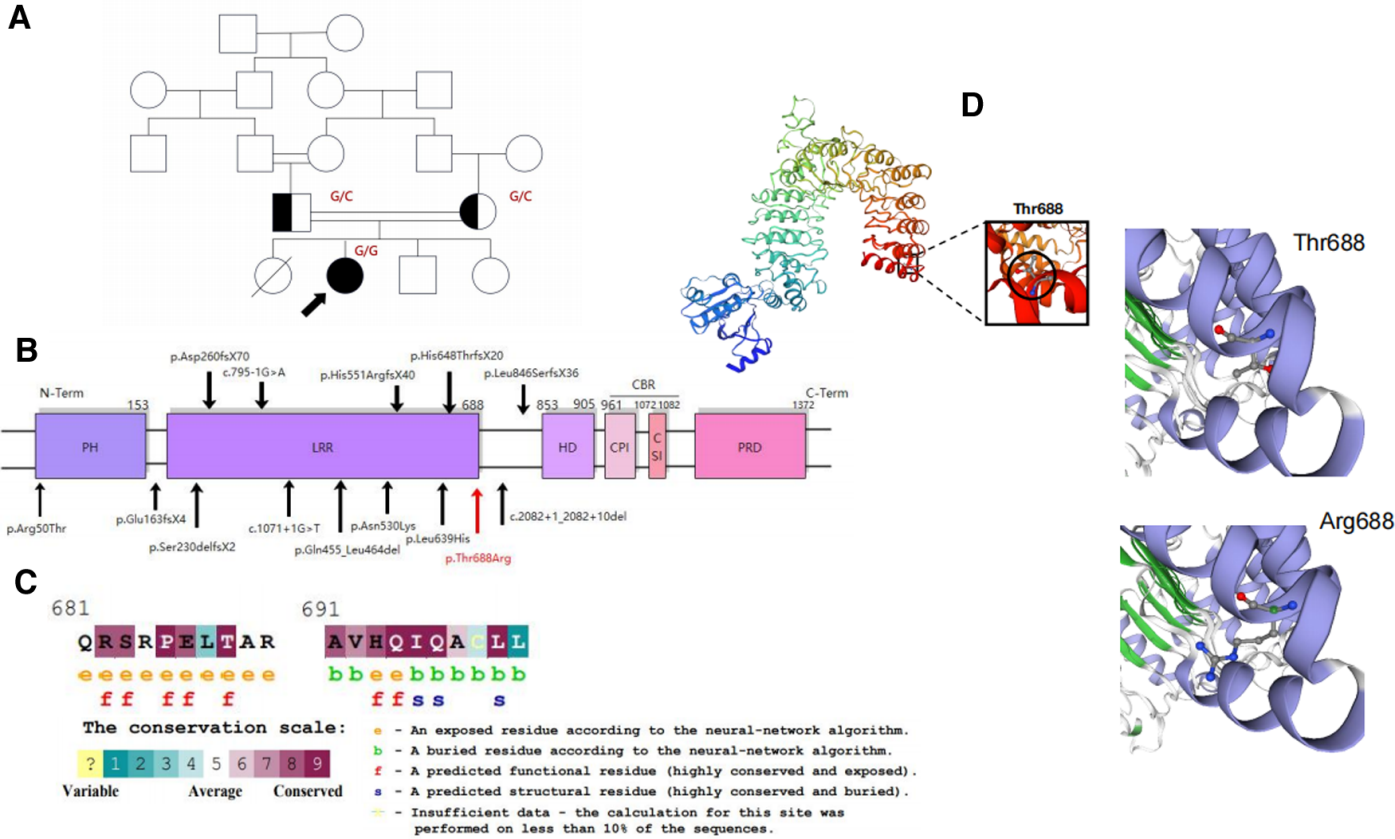
(**A**) Pedigree of the consanguineous families in our study. (**B**) Schematic representation of the CARMIL2 protein domain and its reported variants shown in black. The newly identified variant is shown in red. (**C**) Thr688 is within a highly conserved region near LRR16. It is predicted to be an exposed and functional residue. (**D**) Three-dimensional modeling of CARMIL2 with the location of p. Thr688 established by SWISS-MODEL.

Because of the recurrent skin and mucosal inflammation, immunodeficiency or autoinflammatory diseases and autoimmune diseases were considered possible diagnoses. Thus, impetiginized atopic dermatitis, contact dermatitis, autoimmune pemphigus, or other immunodeficiencies were considered. Behcet's disease was also considered, given the patient's history of recurrent oral ulcers. Immunophenotyping of the patient demonstrated decreased absolute T-cell, B-cell, and NK cell counts but normal regulatory T-cell counts initially. Allergen testing revealed no allergens. The immunologic evaluation included measurements of immunoglobulin levels, antinuclear antibodies, and anti-double-stranded DNA (anti-dsDNA), all of which were initially negative. In addition, EBV-DNA and cytomegalovirus (CMV)-DNA quantity was within normal limits, excluding EBV or CMV infection. Gastrointestinal endoscopy showed evidence of mild chronic colitis with a scattered accumulation of eosinophils. Interstitial lung vasodilation and congestion were observed with an accompanying infiltration of a large number of scattered and focal inflammatory cells in the lung biopsy sample taken in August 2020. Acute inflammatory cells and tissue cells were also observed in the bronchioles. The patient had an older sister that died at a young age due to a fatal respiratory infection and two healthy siblings (Figure 1A).

The patient was treated with corticosteroids, hydroxychloroquine, mycophenolate mofetil, and thymosin for 2 years. The initial dose of prednisone was 15 mg per day (0.6 mg/kg) in July 2020, and hydroxychloroquine (0.1 g per day) and thymosin (1.6 mg biw) were used at the same time. Under this multitargeted treatment, many of her clinical symptoms were relatively improved. The most significant changes included the regression of rashes, especially perioral and perineum rashes, and the improvement of otitis media. Dosages of prednisone were gradually reduced to 2.5 mg every other day in July 2021. However, the patient still suffered from an intermittent cough and recurrent oral ulcer. In addition, the alveolar septal fibrosis caused by recurrent lung infections was not significantly improved. Considering the frequent recurrence of oral ulcers and intermittent cough, the patient had to take 5 mg of prednisone every other day in August 2021, and the dose gradually increased to 10 mg per day in November 2021. Meanwhile, mycophenolate mofetil (250 mg bid) was also given. The patient has been treated with prednisone (7.5 mg per day) and hydroxychloroquine (0.1 g per day) to prevent the recurrence since August 2022.

### Genetic workup

To exclude the possibility of genetic diseases, whole exome sequencing was performed on the patient and her parents, which revealed an unreported homozygous missense variant in CARMIL2 (NM_001013838.2:c.2063C>G:p.Thr688Arg) that was predicted to be deleterious in the patient. Both parents were asymptomatic carriers of the heterozygous variant of CARMIL2. The missense CARMIL2 variant found in the patient was located 1-bp downstream of an LRR16 domain spectrum and had not been previously reported in the general population variant databases (Figure 1B, Supplementary Table S1). The mutation was also highly conserved between species (Figure 1C).

### Identification of the unreported CARMIL2 missense variant

To explore the effects of the missense variant in the CARMIL2 gene on mRNA and protein levels, PBMCs were isolated from the patient and her heterozygous family members.

**Figure 2:**
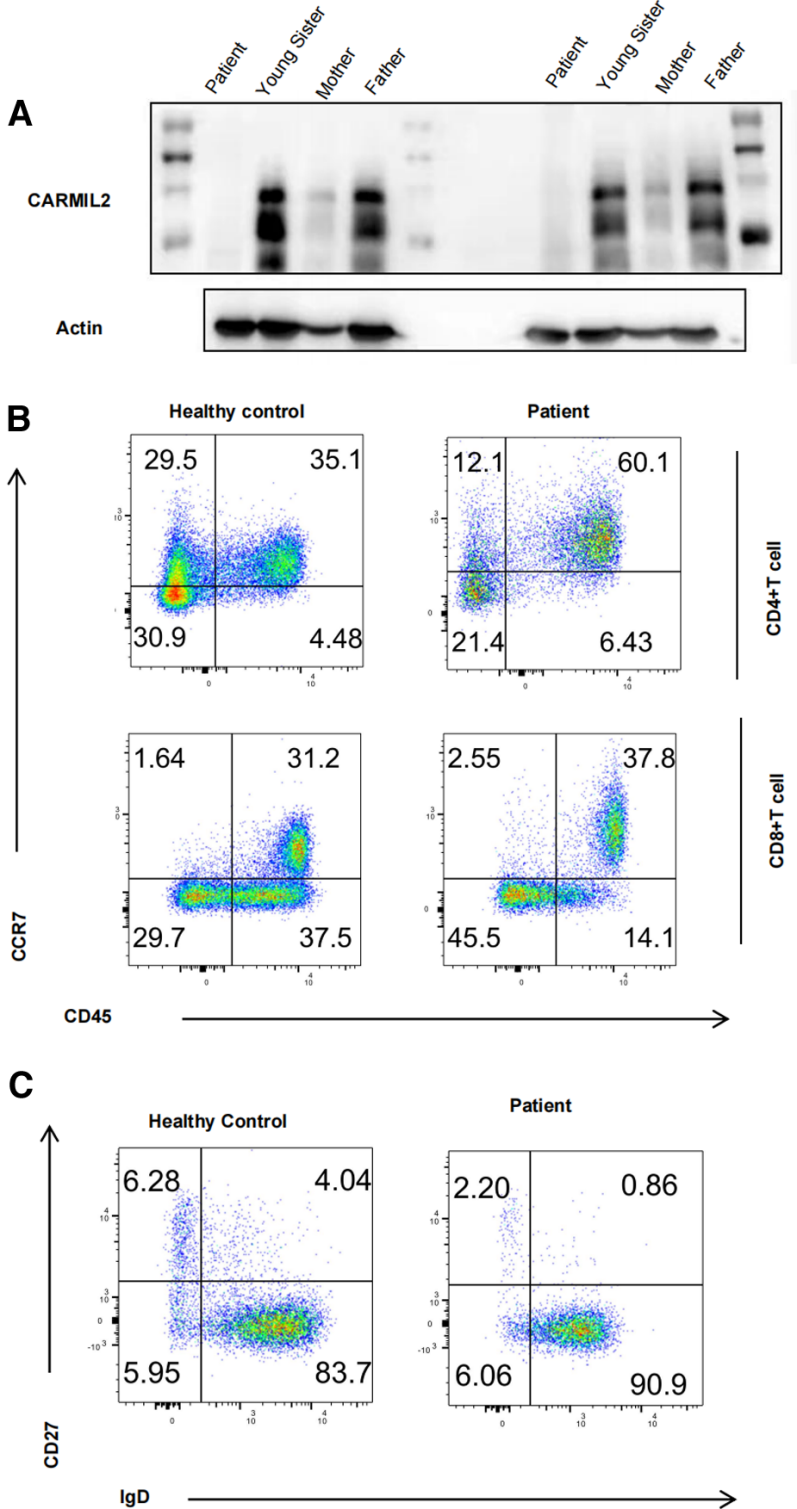
(**A**) Immunoblot analysis of our patient and her heterozygous family members. (**B**) Graphs show CD4/CD8 T-cell subtype populations. The contour plot shows that CD4 and CD8 T cells that are naïve (TN, CD45RA^+^CCR7^+^), central memory (T_CM_, CD45RA^−^CCR7^+^), and effector memory (T_EM_, CD45RA^−^CCR7^−^) T cells (corresponding percentages are indicated in each square). (**C**) Contour plot showing that B cells that are naïve (B_N_, IgD^+^CD27^−^), switched memory (B_SM_, IgD^−^CD27^+^), and nonswitched memory (B_NSM_, IgD^+^CD27^+^) B cells (corresponding percentages are indicated in each square).

Western blot results showed that serum CARMIL2 protein levels were significantly lower in the patient compared to protein levels in the patient's parents and younger sister (Figure 2A), indicating that the missense variation in the CARMIL2 gene results in insufficient expression of CARMIL2.

### 3D homology modeling of the structure of CARMIL2

The CARMIL2 protein consists of an N-terminal noncanonical pleckstrin homology (PH) domain, an LRR domain, a helical homodimerization domain (HD), an extended intrinsically disordered region that contains the capping protein-binding region (CBR), and a proline-rich domain (PRD) that interacts with the SH3 domains of class-I myosin (4, 21) (Figure 1B). The LRR domain is divided into 16 parts (6) that are necessary and sufficient for localization to vimentin. The LRR domain structure consists of repeating regions with a β-strand-turn-α-helix structure and a horseshoe sharp with a solvent-accessible concave interior surface made of parallel β-strands and a convex exterior surface made of an array of α-helices (8, 21, 22). The CBR is composed of two conserved motifs, the capping protein (CP) interaction (CPI) motif (23), which has the ability to decrease the affinity of CP for actin filaments (8) and the CARMIL-specific interaction (CSI) motif (1), which plays a role in inhibiting actin capping *via* CP binding.

A homology model of the horseshoe sharp LRR region was constructed using the SWISS-MODEL template library (24), and the variant lies adjacent to LRR16 (total 16). Exchanging an uncharged amino acid for another considerably larger, positively charged side chain may destabilize the surrounding structure of the protein and hence disrupt the function of the protein (Figure 1D).

### Immunological analyses

An immunologic investigation was performed on the patient, including detailed T-cell, regulatory T-cell (Treg), and B-cell immunophenotyping to evaluate the immune status before and during treatment. Initially, the patient's immunophenotype showed that absolute T-cell and NK cell counts were reduced, but regulatory T-cell counts were normal (Table 1). CD19+ B-cell counts and immunoglobulin levels did not exhibit significant deviation except IgA. Interestingly, the levels of interleukin-17α (IL-17α) and interferon-γ (IFN-γ) were significantly decreased.

**Table 1:** Immune workup of the patient.

Laboratory	Patient's initial value	Normal range
Lymphocyte subset quantification		
CD16+/CD56+ NK cells (cells/µl)	3.27	90–900
CD16+/CD56+ NK cells (%lymphocytes)	3.63%	4%–26%
CD3+/CD4+ T cells (cells/µl)	10.34	300–2,000
CD3+/CD4+ T cells (%lymphocytes)	11.49%	27%–53%
CD3+/CD8+ T cells (cells/µl)	49.96	300–1,800
CD3+/CD8+ T cells (%lymphocytes)	55.51%	19%–34%
CD19+/CD20+ B cells (cells/µl)	640.95	200–1,600
CD19+/CD20+ B cells (%lymphocytes)	15.9%	10%–31%
	Patient	Mean of 17 HCs
T-cell subsets		
CD4+CD45RA−CCR7+ (CD4+T CM)%	12	19.01
CD4+CD45RA+CCR7+ (CD4+T N)%	55.6	54.69
CD4+CD45RA−CCR7− (CD4+T EM)%	25.7	17.12
CD4+CD45RA+CCR7− (CD4+T E)%	6.7	9.18
CD8+CD45RA−CCR7+ (CD8+T CM)%	2.57	3.05
CD8+CD45RA+CCR7+ (CD8+T N)%	36.9	50.43
CD8+CD45RA−CCR7− (CD8+T EM)%	46.1	24.75
CD8+CD45RA+CCR7− (CD8+T E)%	14.4	21.79
B-cell subsets		
CD19+CD27+IgD− (switched memory B-cell)%	2.2	8.22
Regulatory T-cell quantification		
CD4+FOXP3+ cells	0.24/µl	—
CD4+FOXP3+ cells (%lymphocytes)	0.26%	—
B-cell subset phenotyping		
IgG (g/L)	11.6	6.7–15.3
IgM (g/L)	1.67	0.48–2.31
IgA (g/L)	3.5	0.52–2.74
IgE (IU/ml)	<4.23	<200
Complement C3 (g/L)	1.53	0.9–1.8
Complement C4 (g/L)	0.35	0.1–0.4
Others		
IL-2 (pg/ml)	2.18	0.64–8.84
IL-4 (pg/ml)	1.01	0.1–3.88
IL-6 (pg/ml)	1.43	1.05–15.8
IL-10 (pg/ml)	0.89	0.45–4.98
IL-17 (pg/ml)	8.29	16.67–65.76
TNF-α (pg/ml)	1.32	0.1–5.97
Interferon-γ (pg/ml)	0.00	0.44–16.2

Combined therapy was initiated and maintained for 2 years. Following the treatment, the patient's immune phenotypes were assessed again. As listed in Table 2, CD4+ T-cell, CD8+ T-cell, and NK cell counts were significantly improved. Certainly, the long-term clinical effects were still observed.

**Table 2:** Immune workup before and after treatment.

Laboratory	Patient's initial value	Patient's post-treatment value	Normal range
Lymphocyte subset quantification			
CD16+/CD56+ NK cells (cells/µl)	3.27	112.38	90–900
CD16+/CD56+ NK cells (%lymphocytes)	3.63%	2.88%	4%–26%
CD3+/CD4+ T cells (cells/µl)	10.34	1,385.05	300–2,000
CD3+/CD4+ T cells (%lymphocytes)	11.49%	35.44	27%–53%
CD3+/CD8+ T cells (cells/µl)	49.96	1,270.70	300–1,800
CD3+/CD8+ T cells (%lymphocytes)	55.51%	32.52%	19%–34%
CD19+/CD20+ B cells (cells/µl)	640.95	732.45	200–1,600
CD19+/CD20+ B cells (%lymphocytes)	15.9%	18.74%	10%–31%
Others			
IL-2 (pg/ml)	2.18	4.43	0.64–8.84
IL-4 (pg/ml)	1.01	<2.44	0.1–3.88
IL-6 (pg/ml)	1.43	14.32	1.05–15.8
IL-10 (pg/ml)	0.89	5.39	0.45–4.98
IL-17 (pg/ml)	8.29	6.51	16.67–65.76
TNF-α (pg/ml)	1.32	13.00	0.1–5.97
Interferon-γ (pg/ml)	0.00	27.22	0.44–16.2

To access the immunological function of CARMIL2-deficient T cells, PBMCs were extracted from the patient and one age-matched control and then gated and stained with CD45RA/CCR7. As mentioned in previous research (5, 12, 13, 18), T-cell activation was impaired. CD4 T cells exhibited significantly larger differences in the percentage of cells compared to CD8 T cells. The marked skewing to the naïve form indicates defective maturation of T cells (Figure 2B). Meanwhile, marked decreases in IgD^−^CD27^+^ switched memory (B_SM_) and IgD^+^CD27^+^ nonswitched memory (B_NSM_) cells were observed and suggested impaired maturation of B cells in CARMIL2-deficient patients (Figure 2C). Further functional research like lymphocyte proliferation is warranted in the future.

## Discussion

In this article, we reported a female patient from a consanguineous Chinese family with an unreported pathogenic variant in CARMIL2 (c.2063C>G:p.Thr688Arg) who presented with various symptoms of PID. These symptoms included recurrent upper and lower respiratory infections, perioral and perineum papules, erythematous impetiginized atopic dermatitis, oral ulcer, painful urination and vaginitis, otitis media, and failure to thrive. This missense mutation led to insufficient CARMIL2 protein expression, reduced absolute T-cell and NK cell counts, and marked skewing of the naïve T-cell form, indicating defective maturation of T cells and B cells. This is the first report of a new, unrecorded CARMIL2 variant of Chinese descent, expanding the clinical spectrum of CARMIL2 deficiency.

Summarizing previously published clinical characteristics associated with different CARMIL2 mutations showed that the clinical manifestations of the different mutations appear to be heterogeneous. The patient in the current report had a striking feature of cutaneous and respiratory infections, consistent with most of the previous findings associated with different CARMIL2 mutations (5, 12–17, 19, 20). Notably, as listed in Supplementary Table S1, several studies (5, 12, 13, 25) have mentioned that EBV infection or EBV-triggered lymphoproliferative disorders may be a prominent finding in CARMIL2-deficient patients. To explore this point, EBV and CMV quantification and gastrointestinal endoscopy were performed on the present patient, but there was no evidence of EBV/CMV infection or EBV+ smooth muscle tumor. More examinations, such as a positron emission tomography-computed tomography (PET-CT) scan, may provide more information regarding EBV/CMV infection or associated tumors. In addition, the current patient did not suffer from gastrointestinal problems, such as inflammatory bowel disease (IBD), chronic diarrhea, esophagitis, or dysphagia, that have often been observed in patients with other CARMIL2 mutations (12, 15–19). The mechanisms by which different CARMIL2 variants spread along the gene may lead to different immune phenotypes and remains poorly understood.

To estimate the pathogenic effects of the current CARMIL2 mutation, the bioinformatic SWISS-MODEL tool was used to predict the mutant CARMIL2 structure. The resulting 3D model revealed an exchange of the uncharged amino acid Thr for another considerably larger, positively charged side chain, potentially destabilizing the surrounding structure of the protein and disrupting the function of the protein. Additionally, the exchange of charge may generate perturbations in the electrostatic potential distribution (13), which has been shown in another study with a reversed exchange in the LRR1 domain of the same CARMIL2 gene (Figure 1D). Thus, the exchange of the amino acid was likely related to the decrease in CARMIL2 expression in the patient's PBMCs. More specific mechanisms should be addressed in future studies.

The mechanism by which the mutation at site 688 led to the downregulation of CARMIL2 expression was proposed according to the 3D model that was generated. There are two main hypotheses. On the one hand, the LRR domain has an essential role in CD28 costimulation by blocking colocalization with CARMA1 at the immune synapse (9). The initiation, formation, and maintenance of immunocyte synapses rely upon the polymerization and dynamic rearrangement of the cortical actin cytoskeleton (26). As a result, deficiency of CARMIL2 may lead to remodeling of the cortical actin cytoskeleton on T cells and subsequent activation by altering its colocalization with CARMA1. Thus, CARMIL2 is required for CD28 cosignaling in T cells for subsequent maturation and function (7, 8). Regarding naive CD4+ T cells, CARMIL2 deficiency impairs the differentiation of naive CD4+ T cells, leading to the decrease of Th1 and Th17 production, IFN-γ, and IL-17α (2, 27).

On the other hand, the LRR domain is necessary for colocalization with the vimentin intermediate actin filament network (6). There is a list of PIDs associated with actin-related cytoskeletal defects (28). Vimentin, which is filled with dynamic actin filament networks nucleated by the Arp2/3 complex (29, 30), is important for collective cell migration based on wound healing. The current research indicated that the p.Thr688Arg variant is localized adjacent to LRR16, affecting the “horseshoe sharp” structure and changing the electrostatic potential distribution and interaction with vimentin. CARMIL2 may act as a critical link connecting vimentin to the migration, invasion, and wound healing of lymphocytes (31, 32). Interestingly, the vimentin network is functionally “upstream” to CARMIL2, whereas the actin network is functionally “downstream” (6). Lanier et al. (6) depleted CARMIL2 and found that vimentin filament networks were not affected, although F-actin was disrupted. CARMIL2 regulates CP, which is a critical determinant of actin assembly and actin-based cell motility (6). Actin polymerization plays a pivotal role in the formation of the immunological synapse, antigen recognition, signal transduction, and T-cell proliferation, migration, adhesion, and invasion into tissues during the immune response (33, 34). Hence, CARMIL2 deficiency inhibits Arp2/3-dependent actin network assembly by regulating CP at the leading edge and inhibiting barbed-end capping (35), affecting the immune response.

In addition, in the present study, the patient's B-cell maturation was impaired. Most research on B-cell maturation has focused on investigating the proliferation and differentiation of T cells. Wang et al. (7) described biallelic loss-of-function mutations in CARMIL2, affecting the CD28-responsive pathway and B-cell receptor (BCR)-responsive pathway in B cells. More experiments are needed to explore how B-cell function is affected.

Because no specific treatment directly targeting the impaired immune pathway has been established yet, the clinical treatment of PID due to CARMIL2 deficiency is extremely limited. At present, the patient still needs a small dose of prednisone (7.5 mg per day) to prevent the recurrence, which may be due to the mountainous environment and limited local medical care. We consider that the immune dysregulated phenotype is rescued by immunomodulation. Allogeneic hematopoietic stem cell transplant (Allo-HSCT) is a potential treatment for CARMIL2 deficiency (16, 36); however, the lack of long-term data makes it impossible to use Allo-HSCT as a routine treatment for CARMIL2 deficiency. Moreover, when immunophenotypes were assessed after treatment, CD4+ T cells, CD8+ T cells, and NK cells exhibited significant increases (Table 2). The mechanisms of this immunotherapy are still unclear, and further experiments are needed.

## Conclusion

Here, we described a patient with primary immunodeficiency who presented with prominent cutaneous and respiratory infections and in which an unreported homozygous variant in CARMIL2 was identified. The mutation led to decreased protein expression and T-cell activation and proliferation, which were manifested clinically. The clinical characteristics of the patient broadened the spectrum of PID symptoms, highlighting the importance of genetic diagnosis in patients with PID. Because the initial phenotype of the patient could be rescued partially by immunotherapy, the present report provides clinical treatment for similar patients.

## Data Availability

The original contributions presented in the study are included in the article/Supplementary Material, further inquiries can be directed to the corresponding author.
