# Supplementary material for: Case report: Primary immunodeficiency due to a novel mutation in CARMIL2 and its response to combined immunomodulatory therapy
Source: Front Pediatr. 2023 Jan 16;10:1042302. doi: 10.3389/fped.2022.1042302 (PMC9884805; doi:10.3389/fped.2022.1042302)
Supplement: Supplementary file 1 [file Datasheet1.pdf]

Laboratory	Patient's Initial Value	Normal range
**Lymphocyte subset quantitation**		
CD16+/CD56+ NK cells	3.27 cells/ul	90-900 cells/ul
CD16+/CD56+ NK cells (%lymphocytes)	3.63%	4-26%
CD3+/CD4+ T cells	10.34 cells/ul	300-2000 cells/ul
CD3+/CD4+ T cells (%lymphocytes)	11.49%	27-53%
CD3+/CD8+ T cells	49.96 cells/ul	300-1800 cells/ul
CD3+/CD8+ T cells (%lymphocytes)	55.51%	19-34%
CD19+/CD20+ B cells	640.95 cells/ul	200-1600 cells/ul
CD19+/CD20+B cells (%lymphocytes)	15.9%	10-31%
**T cell subsets**	**Patient**	**Mean of 17 HCs**
CD4+CD45RA-CCR7+(CD4+T CM)%	12	19.01
CD4+CD45RA+CCR7+(CD4+T N)%	55.6	54.69
CD4+CD45RA-CCR7-(CD4+T EM)%	25.7	17.12
CD4+CD45RA+CCR7-(CD4+T E)%	6.7	9.18
CD8+CD45RA-CCR7+(CD8+T CM)%	2.57	3.05
CD8+CD45RA+CCR7+(CD8+T N)%	36.9	50.43
CD8+CD45RA-CCR7-(CD8+T EM)%	46.1	24.75
CD8+CD45RA+CCR7-(CD8+T E)%	14.4	21.79
**B cell subsets**		
CD19+CD27+IgD-(switched memory B cell)%	2.2	8.22
**Regulatory T-cell quantitation**		
CD4+FOXP3+ cells	0.24/ul	--
CD4+FOXP3+ cells (%lymphocytes)	0.26%	--
**B-cell subset phenotyping**		
IgG	11.6 g/L	6.7-15.3 g/L
IgM	1.67 g/L	0.48-2.31 g/L
IgA	3.5 g/L	0.52-2.74 g/L

IgE	＜4.23 IU/ml	＜200 IU/ml
Complement C3	1.53 g/L	0.9-1.8 g/L
Complement C4	0.35 g/L	0.1-0.4 g/L
**Others**		
IL-2	2.18 pg/ml	0.64-8.84 pg/ml
IL-4	1.01 pg/ml	0.1-3.88 pg/ml
IL-6	1.43 pg/ml	1.05-15.8 pg/ml
IL-10	0.89 pg/ml	0.45-4.98 pg/ml
IL-17A	8.29pg/ml	16.67-65.76pg/ml
TNF	1.32 pg/ml	0.1-5.97 pg/ml
γ interferon	0.00 pg/ml	0.44-16.2 pg/ml
