# Supplementary material for: Case report: Primary immunodeficiency due to a novel mutation in CARMIL2 and its response to combined immunomodulatory therapy
Source: Front Pediatr. 2023 Jan 16;10:1042302. doi: 10.3389/fped.2022.1042302 (PMC9884805; doi:10.3389/fped.2022.1042302)
Supplement: Supplementary file 2 [file Datasheet2.pdf]

Laboratory	Patient's Initial Value	Patient's Post-treatment outcome	Normal range
**Lymphocyte subset quantitation**			
CD16+/CD56+ NK cells	3.27 cells/ul	112.38 cells/ul	90-900 cells/ul
CD16+/CD56+ NK cells (%lymphocytes)	3.63%	2.88%	4-26%
CD3+/CD4+ T cells	10.34 cells/ul	1385.05 cells/ul	300-2000 cells/ul
CD3+/CD4+ T cells (%lymphocytes)	11.49%	35.44	27-53%
CD3+/CD8+ T cells	49.96 cells/ul	1270.70 cells/ul	300-1800 cells/ul
CD3+/CD8+ T cells (%lymphocytes)	55.51%	32.52%	19-34%
CD19+/CD20+ B cells	640.95 cells/ul	732.45 cells/ul	200-1600 cells/ul
CD19+/CD20+B cells (%lymphocytes)	15.9%	18.74%	10-31%
**Others**			
IL-2	2.18 pg/ml	4.43 pg/ml	0.64-8.84 pg/ml
IL-4	1.01 pg/ml	＜2.44 pg/ml	0.1-3.88 pg/ml
IL-6	1.43 pg/ml	14.32 pg/ml	1.05-15.8 pg/ml
IL-10	0.89 pg/ml	5.39 pg/ml	0.45-4.98 pg/ml
IL-17A	8.29pg/ml	6.51 pg/ml	16.67-65.76pg/ml
TNF	1.32 pg/ml	13.00 pg/ml	0.1-5.97 pg/ml
γ interferon	0.00 pg/ml	27.22 pg/ml	0.44-16.2 pg/ml
