# Supplementary material for: Case report: Primary immunodeficiency due to a novel mutation in CARMIL2 and its response to combined immunomodulatory therapy
Source: Front Pediatr. 2023 Jan 16;10:1042302. doi: 10.3389/fped.2022.1042302 (PMC9884805; doi:10.3389/fped.2022.1042302)
Supplement: Supplementary file 3 [file Datasheet3.pdf]

**General information**	**1**	**2**	**3**
Patient number	3 families, 7 patients	2 families, 4 patients	2 families, 4 patients
Origin	F1:P1-P3: Saudi/Germany	F1:P1-P2 Yemenite/ Germany	Arab/Israel
	F2:P1-P2: Saudi/Germany	F2: P1-P2 Brazillian Germany	
	F3:P1-P2: Saudi/Germany		
Consanguinity	Yes	Yes	Yes
Variants	CARMIL2 (c.2536_2548del:p.L846SfsX36)(1)	CARMIL2(c. 489insG:p.E163fsX4)(2)	CARMIL2(c.A689_714 del: p.S230del-fsX2)(3)
	CARMIL2 (c.2536_2548del:p.L846SfsX36)	CARMIL2(c.871 +1G>T:p.D260fsX70)	
	CARMIL2(c.149G>C:p.Arg50Thr)		
Published time	2018.02	2017.01	2020.06
**Clinical presentations**			
Cutaneous infection	Persistent dermatitis	Recurrent skin abscess	CMV/HPV infection with warts
Mucosal infection	Recurrent skin abscess	Eczematous dermatitis	
	Eczematous dermatitis	Skin warts	
	Scalp Aspergillus niger infection	Recurrent chickenpox	
	Disseminated skin warts		

Chest/respiratory infections<br>Bronchiectasis	Recurrent chest infection<br>Secondary bronchiectasis	Recurrent upper airway infections<br>Recurrent pneumonia	
Dysphagia<br>Lymphocytic esophagitis	Chronic active esophagitis and esophageal candidiasis<br>Dysphagia		Lymphocytic esophagitis
Diarrhea/IBD		Chronic diarrhea	Crohn's disease<br>Ulcerative pancolitis
Lymphadenitis/<br>Generalized lymphadenopathy	Cervical lymphadenitis		
EBV viremia/EBV+ smooth muscle tumor or other tumors	EBV viremia	EBV+ smooth muscle tumor	EBV viremia
Otitis media<br>Nasal vestibulitis<br>Eye ptosis	Persistent otitis media<br>Nasal vestibulitis		
Failure to thrive		Failure to thrive	
Poor response to antigen			

(Continued)

General information	4	5	6
Patients	1 Family, 3 patients	1 family, 1 patient	1 family, 2 patients
Origin	Not mentioned	Syrian/Germany	Saudi Arabian/Saudi Arabia
Consanguinity	Yes	Yes	Yes
Variants	CARMIL2 (c.795-1G>A:p.Met227*)(4)	CARMIL2 (c.1071+1G>T)& (5)	CARMIL2(c.1364_1393del:p.Gln455_Leu464del)(6)
		PLEC1(c.7468C>T:p.Gln2490*)	DOCK8(c.4268C>G:p.Ala1423Gly)&
			DOCK8(c.1306C>T:p.Arg436Trp)
Published time	2019.04	2019.11	2020.06
**Clinical presentations**			
Cutaneous infection	Disseminated warts	Eczematous skin lesions	Eczema
Mucosal infection	Molluscum	Dystrophic nails	
	Verrucous papules		
	Perianal/gluteal Mollusca contagiosa		
Chest/respiratory infections/Bronchiectasis		Recurrent respiratory infections	Asthma
Dysphagia/Lymphocytic esophagitis			
Diarrhea/IBD		Chronic diarrhea	Crohn’s disease

Lymphadenitis/<br>Generalized lymphadenopathy		Generalized lymphadenopathy	
EBV viremia/EBV+ smooth muscle tumor or other tumors			Simultaneous tumors (L5-S1 para-spinal region, lungs, colon, gallbladder, kidneys)<br>Right occipital meningioma
Otitis media/Nasal vestibulitis/Eye ptosis			Right eye ptosis
Failure to thrive		Failure to thrive	Growth hormone deficiency
Poor response to antigen			

(Continued)

**General information**	**7**	**8**	**9**
Patients	1family, 1 patient	3 families, 5 patients	3 families, 4 patients
Origin	Arab/ America	P1-P2: Arab/Russia<br>P3: South Asia/India<br>P4-P5: Turkish/Turkey	Norwegian/Norway
Consanguinity	Not mentioned	Yes<br>Not mentioned	Not mentioned

		Yes	
Variants	CARMIL2(c.1590C>A:p.Asn530Lys)(7)	CARMIL2 (c.2082+1_2082+10del)(8)<br>CARMIL2 (c.1652-_1653delAT:p.H551RfsX40)<br>CARMIL2 (c.688_689delAG:p.Ser230ProfsX2)	CARMIL2(c.1916T>A:p.Leu639His)(9)
Published time	2019.05	2019.10	2016.09
**Clinical presentations**			
Cutaneous infection	Psoriatic rash		Warts
Mucosal infection	Aphthous stomatitis		Eczema<br>Widespread molluscum contagiosum<br>Psoriatic lesions and seborrheic dermatitis<br>Recurrent condyloma<br>UVA sensitive dermatitis with solar urticaria
Chest/respiratory infections			Asthma
Bronchiectasis			Recurrent respiratory infections

			Chronic obstructive pulmonary disease
Dysphagia/Lymphocytic esophagitis			
Diarrhea/IBD	Chronic bloody diarrhea	Pancolitis<br>Mucous diarrhea<br>Abdominal pain	Necrotizing enterocolitis<br>Gastric ulcer& duodenal ulcer<br>Chronic diarrhea and abdominal pain<br>Crohn's disease<br>Recurrent mouth ulcers<br>Gastrointestinal bleeding and diverticulosis
Lymphadenitis/Generalized lymphadenopathy			Hemophagocytic lymphohistiocytosis
EBV viremia/EBV+ smooth muscle tumor			
Otitis media/Nasal vestibulitis/Eye ptosis			
Failure to thrive	Severe failure to thrive<br>Malnutrition<br>Subsequent physical and motor developmental delay	Failure to thrive	Short stature

Poor response to antigen	
**General information**	**10**
Patients	3family, 4patient
Origin	Patient1-2: Moroccan/Italy
	Patient3-4: Not mentioned
Consanguinity	Family1: Yes
	Family2-3: Not mentioned
Variants	CARMIL2(c.462delC:p.Cys155ValfsTer54)(10)
	CARMIL2(c.2932G＞T:p.Glu978*)
	CARMIL2(c.1869C＞A:p.Asp623Glu)
Published time	2021.03
**Clinical presentations**	
Cutaneous infection	Eczema
Mucosal infection	
Chest/respiratory infections	Reactive airway disease
Bronchiectasis	

Dysphagia/Lymphocytic esophagitis	
Diarrhea/IBD	IBD
Lymphadenitis/Generalized lymphadenopathy	
EBV viremia/EBV+ smooth muscle tumor	
Otitis media/Nasal vestibulitis/Eye ptosis	
Failure to thrive	Developmental delay and behavioral disorders
Poor response to antigen	

REFERANCE

1. Alazami AM, Al-Helale M, Alhissi S, Al-Saud B, Alajlan H, Monies D, et al. Novel CARMIL2 Mutations in Patients with Variable Clinical Dermatitis, Infections, and Combined Immunodeficiency. Frontiers in immunology. 2018;9:203.
2. Schober T, Magg T, Laschinger M, Rohlfs M, Linhares ND, Puchalka J, et al. A human immunodeficiency syndrome caused by mutations in CARMIL2. Nature communications. 2017;8:14209.
3. Shamriz O, Simon AJ, Lev A, Megged O, Ledder O, Picard E, et al. Exogenous interleukin-2 can rescue in-vitro T cell activation and proliferation in patients with a novel capping protein regulator and myosin 1 linker 2 mutation. Clinical and experimental immunology. 2020;200(3):215-27.
4. Atschekzei F, Jacobs R, Wetzke M, Sogkas G, Schroder C, Ahrenstorf G, et al. A Novel CARMIL2 Mutation Resulting in Combined Immunodeficiency Manifesting with Dermatitis, Fungal, and Viral Skin Infections As Well as Selective Antibody Deficiency. Journal of clinical immunology. 2019;39(3):274-6.
5. Maccari ME, Speckmann C, Heeg M, Reimer A, Casetti F, Has C, et al. Profound immunodeficiency with severe skin disease explained by concomitant novel CARMIL2 and PLEC1 loss-of-function mutations. Clinical immunology. 2019;208:108228.
6. Yonkof JR, Gupta A, Rueda CM, Mangray S, Prince BT, Rangarajan HG, et al. A Novel Pathogenic Variant in CARMIL2 (RLTPR) Causing CARMIL2 Deficiency and EBV-Associated Smooth Muscle Tumors. Frontiers in immunology. 2020;11:884.
7. Kurolap A, Eshach Adiv O, Konnikova L, Werner L, Gonzaga-Jauregui C, Steinberg M, et al. A Unique Presentation of Infantile-Onset Colitis and Eosinophilic Disease without Recurrent Infections Resulting from a Novel Homozygous CARMIL2 Variant. Journal of clinical immunology. 2019;39(4):430-9.
8. Magg T, Shcherbina A, Arslan D, Desai MM, Wall S, Mitsialis V, et al. CARMIL2 Deficiency Presenting as Very Early Onset Inflammatory Bowel Disease. Inflammatory bowel diseases. 2019;25(11):1788-95.
9. Sorte HS, Osnes LT, Fevang B, Aukrust P, Erichsen HC, Backe PH, et al. A potential founder variant in CARMIL2/RLTPR in three Norwegian families with warts, molluscum contagiosum, and T-cell dysfunction. Molecular genetics & genomic medicine. 2016;4(6):604-16.
10. Bosa L, Batura V, Colavito D, Fiedler K, Gaio P, Guo C, et al. Novel CARMIL2 loss-of-function variants are associated with pediatric inflammatory bowel disease. Scientific reports. 2021;11(1):5945.
